# Emeritus Professor Colin J Masters (1927–2012)

**DOI:** 10.1007/s13205-012-0094-0

**Published:** 2012-10-11



Colin Masters died on April 6 from cancer, after making outstanding and pioneering contributions to biochemistry and biotechnology in Australia and to the development of Griffith University in SE Queensland as a major research based university.

Colin was born in Taihape, a small town on the north island of New Zealand. In his view, the rural setting was an ideal background for his early childhood, full of friendship and scope for youthful adventures, such as canoeing on the local rivers, and exploring the caves, cliffs and other features of the local farmland. Subsequently, he attended a number of different primary schools (as his parents moved from town to town), and then won a scholarship to King’s College (one of NZ’s leading private schools) for his secondary education. There, in his final year, he was the top science student and gained a scholarship to university. He was also a school prefect, head of a house, played in the first fifteen and the first eleven, and represented the school at athletics, gymnastics and swimming.

Casting back to his early boyhood, Colin was fascinated by science from an early age, and when only 10 years old had set up quite a sophisticated chemistry laboratory in a shed at the rear of his parent’s property, where he spent many hours experimenting. He maintained this interest in science throughout secondary school and university, and graduated from Auckland University with a Master’s degree in science.

His first employment after graduating was as a medical scientist in charge of a biochemical laboratory in Auckland. He was keen to further advance his interest in this subject, and moved to a teaching and research position at the University of Queensland in Brisbane in 1960. He stayed there for 15 years, eventually heading the Department of Biochemistry, before accepting a position as the foundation professor of biochemistry at Griffith University. In subsequent years at Griffith University, he served as Dean of Science, and then as Pro-Vice-Chancellor, and on several occasions Acting Vice Chancellor—all the time maintaining his interest in science, and eventually being the recipient of three doctoral degrees. Among his many other academic roles, he chaired key university wide committees, including the academic, education and research committees, and was responsible for introducing many major educational streams, which are now features of the university’s offerings. He was also a member of the State Government Working Party, which advised on the tertiary entrance system in Queensland, and served on the inter-university Advisory Council, which oversaw the physical and academic development of Bond University.

Many of these aspects of his career were brought into focus at his retirement ceremony in 1992, when he was described by the University Chancellor as a gentleman of science, a man of outstanding scientific achievements, broad international reputation, high academic integrity, excellent administrative abilities, and a calm and efficient style of administration.

In addition to his heavy teaching and administrative duties at the University of Queensland and Griffith University, he still managed to carve out an outstanding career in research. He was internationally recognized as a top researcher in his field; an original thinker, who championed several major paradigm shifts in biomedical science. As such, he was often invited to collaborate in research with leading scientists in other countries, and lauded for his seminal development of major innovatory concepts, which enabled important breakthroughs in areas of contemporary interest such as biotechnology, genetic disease, mammalian growth processes, and medication metabolism. In the pursuance of this research, he published more than 300 research papers in international journals, and was an invited lecturer at many overseas research institutes and universities. He was recognized internationally as a leader in several fields of biochemistry and biotechnology, including mammalian development, molecular biology, protein interactions, peroxisomal biogenesis and metabolic control mechanisms. In addition, Colin was renowned for his expert, calm and successful role as a research postgraduate student and postdoctoral supervisor of many scientists, some of whom hold Professorial and other senior positions within universities and other research organizations in Australia and internationally.

During his career and in his years of retirement, Colin Masters also wrote several books as advanced texts on aspects of his research, a number of which were republished in several languages. His most recent book, ‘DNA and Your Body, What you should know about Biotechnology’, though, was directly aimed at the general public. DNA of course is well known as the molecule that programs our human potential (and as being of central importance in relation to the growth, ageing, individuality, diseases and future status of mankind). Aware of the momentous changes that are occurring in DNA biology and the dramatic effect that these advances have on human health and attitudes (and the potential significance of many of these discoveries to present and future generations), he wrote this book in a form, which was readily comprehensible to a general reader to facilitate a broader understanding of these issues among the general public.

Besides writing books on science in his retirement years, Colin also participated in several areas of community interest. As one example, he made many contributions to the game of bridge in Queensland, writing a definitive history, well known to adherents of the game (‘Mind Games’), and spent several years in administrative roles. Upon his retirement (due to ill health) from the central management committee of the Queensland Bridge Association, for example, he was commended for his years of valuable service to the game by the president of the Australian Bridge Federation, and described as a man of integrity, dignity, character and courage.

In addition to his academic interests, Colin Masters maintained an active interest in sports during the whole of his career. Several years of representative rugby and athletics in New Zealand, were followed by an active interest in tennis and golf in his later years in Australia.

Colin is survived by his wife Josephine, three sons, Greg, Alistair and Scott, and six grandchildren.

Roger S Holmes

July 2012

## Representative Publications of Emeritus Professor Colin Masters

M Hinks and CJ Masters. Developmental changes in ruminant lactate dehydrogenase. Biochemistry 3: 1789–1791 (1964).

RS Holmes, CJ Masters and EC Webb. A comparative study of vertebrate esterase multiplicity. Comparative Biochemistry and Physiology 26: 837–852 (1968).

N Kingsbury and CJ Masters. Molecular weight interrelationship in the vertebrate esterases. Biochimica Biophysica Acta 200: 58–69 (1970).

C Gibson and CJ Masters. On the lactate dehydrogenase of preimplantation mouse ova. FEBS Letters 7: 277–279 (1970).

CJ Masters and RS Holmes. Isoenzymes and ontogeny. Biological Reviews 47: 309–361 (1972).

BJ Kitchen, CJ Masters and DJ Winzor. Effects of lipid removal on the molecular size and kinetic properties of bovine plasma arylesterase. Biochemical Journal 135: 93–99 (1973).

CJ Masters and RS Holmes. Isoenzymes, multiple enzyme forms, and phylogeny. Advances in comparative Physiology and Biochemistry 5: 109–195 (1974).

CJ Masters and RS Holmes. Haemoglobin, Isoenzymes and Tissue Differentiation. North Holland Publishers (1975).

RI Brinkworth, CJ Masters and DJ Winzor. Evaluation of equilibrium constants for the interaction of lactate dehydrogenase isoenzymes with reduced NAD by affinity chromatography. Biochemical Journal 151: 631–636 (1975).

M Don and CJ Masters. On the comparative turnover rates of the lactate dehydrogenase isozymes in rat tissues. International Journal of Biochemistry 7: 215–220 (1976).

CJ Masters and RS Holmes. Peroxisomes: new aspects of the cell physiology and biochemistry of these oxidative organelles. Physiological Reviews 57: 816–882 (1977).

RS Holmes and CJ Masters. Genetic control and ontogeny of microbody enzymes: a review. Biochemical Genetics 16: 171–190 (1978).

TP Walsh, DJ Winzor, FM Clarke, CJ Masters and DJ Morton. Binding of aldolase to actin-containing filaments. Evidence of interaction with the regulatory proteins of skeletal muscle. Biochemical Journal 186: 89–98 (1980).

D Crane, RS Holmes and CJ Masters. Proteolytic modification of the mouse liver catalase. Biochemical and Biophysical Research Communications 104: 1567–1572 (1982).

E Klucis, D Crane and CJ Masters. Sequential alterations in the microlocalization of catalase in mouse liver after treatment with hypolipidemic drugs. Molecular and Cellular Biochemistry 65: 73–82 (1984).

C Masters and D Crane. The role of peroxisomes in lipid metabolism trends in Biochemical Sciences 9: 314-317 (1984).

DI Crane, AC Hemsley and CJ Masters. Purification of peroxisomes from livers of normal and clofibrate-treated mice. Analytical Biochemistry 148: 436–445 (1985).

S Reid and CJ Masters. On the ontogeny and interactions of glyceraldehyde-3-phosphate dehydrogenase. Mechanisms of Ageing and Development 35: 209–219 (1986).

L Humphreys, S Reid and CJ Masters. Evidence for the spatial separation of the binding sites for substrate and for cytoskeletal proteins on the enzyme aldolase. International Journal of Biochemistry 18: 7–13 (1986).

N Chen, DI Crane and CJ Masters. Analysis of the major integral membrane proteins of peroxisomes from mouse liver. Biochim et Biophysica Acta-Biomembranes 945: 135–144 (1988).

DI Crane, J Zamettia and CJ Masters. Alterations in the integrity of peroxisomal membranes in livers of mice treated with peroxisome proliferators. Molecular and Cellular Biochemistry 96: 153–161 (1990).

CJ Masters. Cellular differentiation and the microcompartmentation of glycolysis. Mechanisms of Ageing and Development 61: 11–22 (1991).

W Murrell, D Crane and C Masters. Ontogenic characteristics of cavian aldolase. Mechanisms of Ageing and Development 65: 35–50 (1992).

CJ Masters and DI Crane. On the role of the peroxisome in ontogeny, ageing and degenerative disease. Mechanisms of Ageing and Development 80: 69–83 (1995).

CJ Masters and DI Crane. The peroxisome: a vital organelle. Cambridge University Press (1995).

CJ Masters. Cellular signaling: the role of the peroxisome. Cellular Signaling 8: 197–208 (1996).

CJ Masters. Omega-3 fatty acids and the peroxisome. Molecular and Cellular Biochemistry 165: 83–93 (1996).

CJ Masters. Gluconeogenesis and the peroxisome. Molecular and Cellular Biochemistry 166: 159–168 (1997).

C Masters and D Crane. On the role of the peroxisome in cell differentiation and carcinogenesis. Molecular and Cellular Biochemistry 187: 85–97 (1998).

C Masters. DNA and Your Body: What You Need to Know about Biotechnology. University of NSW Press (2005).

